# Non-Invasive Biomarkers for Earlier Detection of Pancreatic Cancer—A Comprehensive Review

**DOI:** 10.3390/cancers13112722

**Published:** 2021-05-31

**Authors:** Greta Brezgyte, Vinay Shah, Daria Jach, Tatjana Crnogorac-Jurcevic

**Affiliations:** Centre for Cancer Biomarkers and Biotherapeutics, Barts Cancer Institute, Queen Mary University of London, London EC1M 6BQ, UK; g.brezgyte@qmul.ac.uk (G.B.); vinay.shah8@nhs.net (V.S.); d.jach@qmul.ac.uk (D.J.)

**Keywords:** pancreatic ductal adenocarcinoma, systematic review, biomarkers, QUADAS-2, early detection, non-invasive

## Abstract

**Simple Summary:**

Pancreatic ductal adenocarcinoma (PDAC), which represents approximately 90% of all pancreatic cancers, is an extremely aggressive and lethal disease. It is considered a silent killer due to a largely asymptomatic course and late clinical presentation. Earlier detection of the disease would likely have a great impact on changing the currently poor survival figures for this malignancy. In this comprehensive review, we assessed over 4000 reports on non-invasive PDAC biomarkers in the last decade. Applying the Quality Assessment of Diagnostic Accuracy Studies (QUADAS-2) tool, we selected and reviewed in more detail 49 relevant studies reporting on the most promising candidate biomarkers. In addition, we also highlight the present challenges and complexities of translating novel biomarkers into clinical use.

**Abstract:**

Pancreatic ductal adenocarcinoma (PDAC) carries a deadly diagnosis, due in large part to delayed presentation when the disease is already at an advanced stage. CA19-9 is currently the most commonly utilized biomarker for PDAC; however, it lacks the necessary accuracy to detect precursor lesions or stage I PDAC. Novel biomarkers that could detect this malignancy with improved sensitivity (SN) and specificity (SP) would likely result in more curative resections and more effective therapeutic interventions, changing thus the present dismal survival figures. The aim of this study was to systematically and comprehensively review the scientific literature on non-invasive biomarkers in biofluids such as blood, urine and saliva that were attempting earlier PDAC detection. The search performed covered a period of 10 years (January 2010—August 2020). Data were extracted using keywords search in the three databases: MEDLINE, Web of Science and Embase. The Quality Assessment of Diagnostic Accuracy Studies (QUADAS-2) tool was applied for study selection based on establishing the risk of bias and applicability concerns in Patient Selection, Index test (biomarker assay) and Reference Standard (standard-of-care diagnostic test). Out of initially over 4000 published reports, 49 relevant studies were selected and reviewed in more detail. In addition, we discuss the present challenges and complexities in the path of translating the discovered biomarkers into the clinical setting. Our systematic review highlighted several promising biomarkers that could, either alone or in combination with CA19-9, potentially improve earlier detection of PDAC. Overall, reviewed biomarker studies should aim to improve methodological and reporting quality, and novel candidate biomarkers should be investigated further in order to demonstrate their clinical usefulness. However, challenges and complexities in the path of translating the discovered biomarkers from the research laboratory to the clinical setting remain and would have to be addressed before a more realistic breakthrough in earlier detection of PDAC is achieved.

## 1. Introduction

Pancreatic ductal adenocarcinoma (PDAC), which represents approximately 90% of all pancreatic cancers, is an extremely aggressive disease and one of the most lethal cancers. Its incidence almost equals its mortality, and it has a 5-year survival of only around 9% [1,2]. It caused 7800 deaths in the United Kingdom (UK) in 2018 alone, and, according to GLOBOCAN, there were 458,918 new cases of pancreatic cancer and 432,242 pancreatic cancer-related deaths worldwide in the same year [3]. Furthermore, PDAC incidence is steadily increasing [4,5,6,7].

PDAC is considered a silent killer due to a largely asymptomatic course, late clinical presentation, and rapid progression [8], with fewer than 20% of PDAC patients being diagnosed at early, resectable stages I and II [9,10]. Importantly, if PDAC is detected when still a localized disease, the 5-year survival is around 32% [11], but it can approach 70% following resection of incidentally diagnosed stage I tumors [12,13,14]. It is estimated that it takes at least 10 years between initiating mutation and the birth of parental founder [15]; combined with an increased PDAC incidence with age [5], resectable PDAC has likely already been growing for a number of years. We therefore further on use the term “earlier” rather than “early” stage.

To date, there exists no reliable, non-invasive screening test, either molecular or imaging-based, that will allow accurate PDAC detection at an earlier stage in asymptomatic patients. The conventional methods such as computerized tomography, magnetic resonance imaging or endoscopic ultrasonography are expensive and have low sensitivity (SN) and specificity (SP) for detection of small premalignant lesions [16]. Furthermore, the deep anatomic location of the pancreas makes detection of small-localized tumors unlikely during a routine abdominal examination [17].

In contrast, testing for biomarkers in body fluids such as serum, plasma, urine or saliva is minimally invasive, relatively inexpensive and could allow for earlier PDAC diagnosis [18]. The current gold-standard serum biomarker for PDAC is carbohydrate antigen 19-9 (CA19-9), also known as sialylated Lewis (a) antigen. It is a product of exocrine epithelial cells and various mucins secreted by PDAC cells that are normally found on the surface of erythrocytes [19]. The test was approved by the U.S. Food and Drug Administration (FDA) in 2002 [20]. However, it is recommended to be used only for disease monitoring and prognosis, since the performance of CA19-9 as a diagnostic biomarker is limited by poor SN, false-negative results in the individuals with Lewis negative phenotype and increased false positivity in several benign and malignant biliary disorders [21]. Furthermore, CA19-9 is not recommended as a screening marker due to its low positive predictive value (0.5–0.9%) [22,23,24].

The accuracy of any tumor biomarkers assay, including CA19-9, is linked to the concept of threshold, or cut-off [25]. The CA19-9 cut-off values differ significantly between studies due to the lack of an international standard for CA19-9 and the difference in the assay design and instrument utilized [26]. When the two most frequently used assays for measuring CA19-9, Abbott Architect CA 19-9XR and Roche Elecsys CA19-9, were compared, despite being based on the same monoclonal antibody 1116-NS-19-9 [27], the Abbott assay measured significantly lower CA19-9 values than the Roche one in healthy and benign cases [28]. While the upper reference limit of 37 U/mL, reaching mean SN and SP of 81% and 90%, respectively, as first reported by Steinberg [29], is still able to sufficiently differentiate between controls and PDAC, the CA19-9 values obtained by different methods should not be used interchangeably, the method utilized should be clearly stated and the patients should be monitored using the same assay [28,30]. Thus, despite its role as the principle serological test for PDAC, the standardization of the laboratory testing and correct interpretation of high serum CA19-9 levels with the adequately established cut off has not yet been achieved [31]. The search for a new non-invasive PDAC biomarker with superior SN and SP to CA19-9, which will be easily identifiable and robustly measured in a fully standardized fashion in biofluids, is therefore being actively pursued.

Recent advances in molecular techniques have empowered the discovery of novel biomarkers for earlier detection of PDAC, resulting in a significant and constantly growing body of literature. Despite the discovery of thousands of biomarkers, relatively few have been adequately tested and validated, making the assessment of their potential challenging [32]. This sparked an EDRN (The Early Detection Research Network) workshop at National Cancer Institute (NCI) in 2016, where a selection of the most promising studies was reported [33]. A comprehensive update and critically appraised resource of information on potentially useful novel biomarkers for PDAC is, however, needed.

In the present study, we reviewed over 4000 articles and further assessed 49 published studies from the last decade describing biomarkers in serum, plasma, urine and saliva for earlier PDAC detection, in conjunction with serum CA 19-9, when available. We utilized the Quality Assessment of Diagnostic Accuracy Studies tool (QUADAS-2) to assess the quality of the selected studies and highlighted their potential clinical utility and limitations for earlier PDAC detection.

## 2. Methods

Two of the authors (G.B. and V.S.) independently conducted a systematic search of MEDLINE, Web of Science and Embase and assessed all the articles published between January 2010 and August 2020. A robust reproducible search strategy was created which utilized the MESH terms “Pancreas OR Pancreatic” AND “Ductal Adenocarcinoma OR PDAC OR Cancer OR Malignancy OR Adenocarcinoma OR Tumour OR Tumor” AND “Liquid Biopsy OR Biomarkers OR Screening OR Stool OR Markers OR Marker OR Urine OR Saliva OR Blood OR Non-invasive OR Non Invasive OR Fluid Biopsy OR Fluid Phase Biopsy OR Detection OR Diagnosis OR Diagnose OR Screen” to search for the potential biomarkers specific for this disease. The filters were adjusted to show the results for studies in the English language only. The article type was set to only include clinical and observational studies in Medline. In Medline and Embase, titles and abstracts were searched. In Web of Science, only titles were searched. PRISMA guidelines were used to assist in the structuring of this systematic review [34]. The inclusion and exclusion criteria for selecting the studies are listed in Table 1.

Assessment of the methodological quality of reviewed articles was performed according to QUADAS-2, a validated quality assessment tool commonly used to assess the methodology of investigated studies, and as a measure to improve diagnostic accuracy [35]. With QUADAS-2, we were able to appraise three key domains, namely Patient Selection, Index Test and Reference Standard, for both risk of bias and applicability. For the Patients selection, we assessed if the sample groups were well described and if patient spectrum was representative of the disease. We also assessed if the selection of the controls was appropriate, i.e., if, in addition to healthy controls, samples collected from patients with benign pancreatic diseases at risk for progressing to PDAC were also included. Within the Applicability concerns, we assessed if the data for PDAC stages I/II were reported, as we were aiming to review biomarkers for earlier detection of PDAC. For the Index test (biomarker assay), we appraised how well the assay was described and executed and the results interpreted (SN/SP/AUC with 95% confidence intervals (CIs)) and if the data were split into training and validation sets. To increase the validity of the results, QUADAS-2 also recommends that researchers do not know which study group each patient belongs to (“blinding”) in order to reduce the effects of any foreknowledge in the interpretation of the biomarker assay results [36]. QUADAS-2 includes the need to specify a threshold value for the Index test prior to obtaining the results; however, this is not suitable for newly discovered biomarkers, where one of the aims is to determine the appropriate cut-offs with regards to the SN, SP and AUC. For the Reference Standard, we assessed if the study specifies that PDAC diagnosis was confirmed by histology and if the staging was pathological or clinical. However, as biomarker discovery studies are typically performed on retrospectively collected samples with an already established diagnosis, we additionally included a comparison of the new biomarker with CA19-9. Flow and Timing were excluded from our QUADAS-2 scoring, as it was difficult to assess this parameter, i.e., in the majority of the studies, it was unclear in what time frame were the diagnostic biopsy or imaging performed in respect to the sample collection and the biomarker testing.

## 3. Results and Discussion

**Figure 1:**
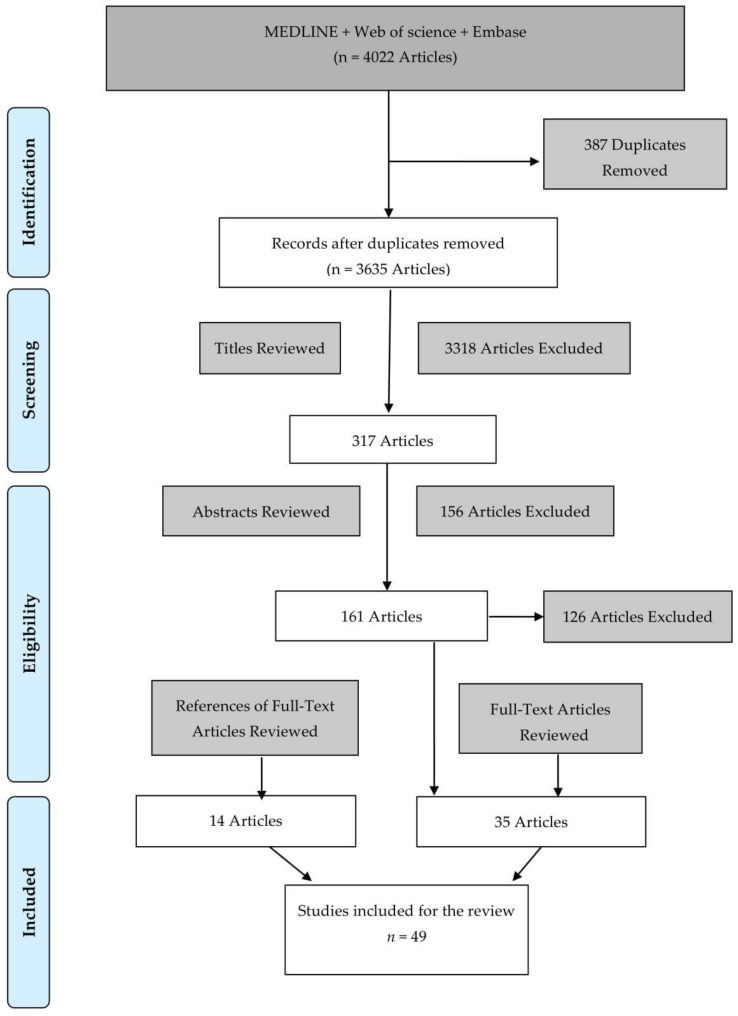
PRISMA Flow diagram of the search strategy and study selection.

A PRISMA flow diagram demonstrating the search strategy and study selection criteria that were applied is shown in Figure 1.

**Figure 2:**
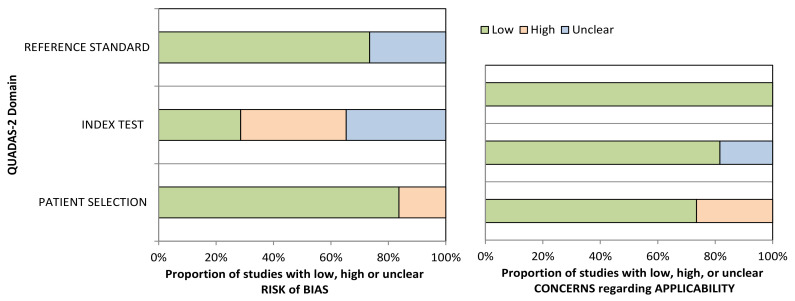
Graphical representation of the overall quality of the selected studies.

The overall quality of the 49 selected and reviewed studies is illustrated in Figure 2.

In Figure 2 it is evident that the majority of the studies (70%) had a low risk of bias in the Reference Standard domain, as the information on histological confirmation that samples originated from PDAC was specified. The unclear bias in this domain seen in approximately 30% of the studies was due to the absence of comparison of biomarkers with CA19-9. High risk of bias in the Index test (biomarker assay) was seen in 36% of studies and was associated with missing information on if the samples were analyzed in the blinded fashion. The lack of clarity, which was seen in the following 36% of the studies, was due to absence of robust statistical analysis of the data, i.e., splitting of the data into the training and the validation sets. Regarding patient selection criteria, around 20% of studies did not compare PDAC results to appropriate benign controls, leading to a high risk of bias. Around 20% of studies raised concerns about the applicability of the biomarkers for earlier PDAC detection, as they were not assessed in resectable stage I–II PDAC.

A graphical summary of the QUADAS-2 results with the descriptive score for each of the selected studies is presented in a tabular form in supplementary Appendix A, Table A1.

Table 2 summarizes the key findings from all 49 studies included in this review.

The studies summarized in Table 2 were all aimed at earlier PDAC detection; however, around 20% of them did not analyze their biomarkers in resectable stage I–II samples. While the paucity of earliest stage I samples is a recognized problem in the PDAC field, this was surprising, considering the critical importance of diagnostic stage shift. Selection of the relevant control population is also of the essence, as PDAC is an uncommon cancer, which dictates the need to assess the putative biomarkers in high-risk groups [1,84,85], a criterion which was not satisfied in 20% of the studies. It also requires the high accuracy of any potential future biomarker assay with at least 88% SN and 85% SP if the test is to be cost-effective [86]. 

A third of the reviewed studies did not include a comparison of their biomarkers to CA19-9, which was unexpected considering that CA19-9 is the most commonly measured biomarker in PDAC patients. Very few biomarkers were conclusively superior to CA19-9 and in the majority of studies where their combination was tested, the accuracy of PDAC detection was superior to either biomarker alone. For example, Dong et al. [71] described the panel of serum biomarkers comprising CA19-9, POSTN and CA242, which was superior to CA19-9 alone in differentiating between PDAC and both healthy controls (AUC of 0.98, 92.1% SN and 97.3% SP) and benign pancreatic conditions (AUC of 0.93, 80% SN and 97.7% SP), albeit with somewhat lower AUC of 0.90 and a decrease in SP to 88% in resectable PDAC. In the study of Berger et al. [77], CA19-9 combined with THBS2 and cfDNA in PDAC vs. non-PDAC patients showed an increase in AUC from 0.70 to 0.78, with an increased SN and SP of 80% and 96%, respectively. A study by Fahrmann et al. [79] combined a five-metabolite panel (acetylspermidine, diacetylspermine, an indole-derivative and two lysophosphatidylcholines) with a previously validated protein panel comprising CA19-9, TIMP1 and LRG1, achieving AUC of 0.924 (95% CI 0.864–0.983). Recently, another panel (FGA, KRT19, HIST1H2BK, ITIH2, MARCH2, CLDN1, MAL2 and TIMP1) obtained by plasma extracellular vesicle long RNA profiling (d-signature), successfully differentiated PDAC from CP and healthy controls with AUC of 0.936, and both SN and SP > 90%; furthermore, when combined with CA19-9, the signature showed improved AUC of 0.964 (95% CI: 0.943–0.984) [80]. Traeger et al., reported that miRNA-205 combined with CA19-9 resulted in higher SN and SP in comparison of PDAC vs. benign specimens (86.7% SN and 93.3% SP) than either of the biomarkers alone [75].

The absolute SN and SP values for PDAC detection using glypican-1 (GPC1) in circulating exosomes when PDAC samples were compared to healthy and benign samples was reported by Melo et al. [54]. However, several subsequent studies failed to support these findings [62,73,87,88], while Zhou et al. [76] demonstrated that combining GPC1 and CA19-9 showed 92.31% SN at 65.83% SP for distinguishing healthy, chronic pancreatitis and benign pancreatic tumors from PDAC. Regardless of the underlying reasons, these conflicting results highlight the critical importance of independent validation of any proposed biomarkers.

The above study [54] and the more recent studies indicate the “expansion” of the biomarker field, moving from the “traditional” DNA, RNA and protein biomarkers to novel, versatile analytes and their combinations, such as non-coding RNAs, epigenetic markers, lipids and metabolites, as well as circulating tumor cells and extracellular vesicles (EV) [88]. For example, Yang et al. [83] performed a multi-parametric analysis of blood tumor-associated EV miRNA and mRNA, circulating cell-free DNA concentration and KRAS G12D/V/R mutations with CA19-9, and demonstrated their higher accuracy for PDAC staging (84%) than imaging alone (accuracy = 64%; P < 0.05). Furthermore, recent advances in computer science, machine learning and artificial intelligence (AI) have given rise to the emerging field of bioinformatics and computational biology which allow analyses of large collections of biological data [89]. While AI was mostly utilized for the prediction of risk/diagnosis based on health records or abdominal imaging [1,90,91], it also shows a great potential for in silico discovery of putative novel biomarkers. For example, Khatri and Bhasin [92] used transcriptomics-based meta-analysis of tissue and blood samples combined with machine learning to identify a nine-gene panel (IFI27, ITGB5, CTSD, EFNA4, GGH, PLBD1, HTATIP2, IL1R2 and CTSA) that differentiated PDAC and healthy controls with 92% SN and 90% SP. Moreover, this panel also discriminated PDAC from chronic pancreatitis and early precursor lesions in non-malignant tissue and peripheral blood. Chung et al. [93] used probe electrospray ionization mass spectrometry (PESI-MS) and a machine learning approach to analyze peripheral blood samples and achieved an accuracy of 92.1% in healthy vs. PDAC (90.8% SN, 91.7% SP). The combination of PESI-MS profiles with age and CA19-9 increased the accuracy for detection of stage I and II PDAC to 92.9% (81.2% SN, 96.8% SP) [40]. This demonstrates that the access to richly annotated patients’ health records will enable the construction of novel PDAC prediction tools such as the one developed by Muhammad et al. [90]. Combining such prediction tools with biofluid biomarkers now opens up promising new avenues and likely a fruitful approach to identify asymptomatic patients at increased risk of developing PDAC.

Very few studies explored the effectiveness of their proposed biomarkers in pre-diagnostic samples from asymptomatic patients. Although such samples are not readily available, these studies are essential for determining how early in the latency period the biomarkers can be detected [40,47,94,95,96,97,98,99,100,101]. Nolen et al. [47] interrogated the large prospective Prostate, Lung, Colorectal, and Ovarian (PLCO) Cancer Screening Trial serum samples to detect eight biomarkers: CEA-related cell adhesion molecule 1 (CEACAM1) and prolactin (PRL) were detectable up to 35 months before PDAC diagnosis; CA19-9, carcinoembryonic antigen (CEA), neuron-specific enolase (NSE) and beta-human chorionic gonadotropin (b-HCG) up to 24 months; and CA125 and interleukin 8 (IL-8) up to 12 months prior PDAC diagnosis. In the UK Collaborative Trial of Ovarian Cancer Screening (UKCTOCS) serum samples, a decrease in circulating TSP-1 levels up to 24 months prior to PDAC diagnosis was described by Jenkinson et al. [97], and O’Brien et al. demonstrated that CA19-9 could be detected in such pre-diagnostic PDAC samples with 95% SP and 68% SN up to 1 year and 53% SN up to 2 years prior to PDAC diagnosis [98]. A three-biomarker panel comprising Erb-B2 receptor tyrosine kinase 2 (ERBB2), estrogen receptor 1 (ESR1) and tenascin C (TNC) was analyzed in 87 pre-diagnostic plasma samples from Women’s Health Initiative (WHI) collected up to 4 years before diagnosis and achieved AUC of 0.71 when combined with CA19-9 [100]. Recently, the analysis of 1196 proteins in longitudinal plasma samples from the Institute for Systems Biology’s 100K Wellness project identified several markers, including three that were observed in PDAC: CEA cell adhesion molecule 5 (CEACAM5) was expressed at high levels 26.5 months pre-diagnosis, while calcitonin related polypeptide alpha (CALCA) and delta1 like homolog (DLK1) were predating PDAC diagnosis for around 17 months [101]. More exciting projects such as this are needed and should be actively encouraged.

While performing this review, we encountered a number of hurdles, particularly noticeable in the way the studies were reported. Easier reviewing of published resources would be facilitated, and the extraction of the relevant information simplified, if a consensus set of standards for the reporting of biomarker studies were utilized. In addition to QUADAS-2, several of such recommendations and guidelines exist: BRISQ (Biospecimen Reporting for Improved Study Quality) [102] provides recommendations for reporting details about the interrogated biospecimens; the Standards for Reporting of Diagnostic Accuracy Studies (STARD) [103] was developed to improve the completeness and transparency of the reports; and Reporting Recommendations for Tumour Marker Prognostic Studies (REMARK) [104] and Consolidated Standards of Reporting Trials (CONSORT Statement) [105] are used to improve the overall quality of tumor markers in the prognostic studies and randomized trials [104,105] . The latter is the final step in establishing the clinical utility of the biomarker, i.e., increased survival due to earlier diagnosis achieved by biomarker testing. Interestingly, only studies published in high-impact factors journals followed any of these recommendations [49,54,66,74,80]. 

In addition to the standardized way of reporting, a standardized pathway for early detection and diagnosis is also needed. Such pathway has recently been summarized in the updated Cancer Research UK (CRUK) roadmap, which aims to consolidate the current diverse and fragmented research activities, in line with the future vision to detect 75% of cancers at stage I-II by 2028 [106]. A roadmap also illustrates a long way from biomarker discovery to its implementation, with a number of hurdles encountered at every step in biomarker pipeline, from initial biomarker discovery, its quantification, verification and assay optimization to finally biomarker validation and commercialization, as comprehensively discussed previously [107,108,109]. While biomarker discovery is usually well funded and results straightforward to report, validation, which is a necessary step prior to commercialization, is time-consuming and expensive process, with scarce funding available. The transition from discovery to the clinical utility of any biomarker is therefore a cumbersome process, akin to the implementation of novel therapeutic [110].

It is not surprising thus that, despite a large number of candidate biomarkers, very few are close to clinical implementation. Based on the accuracy of 95% of serum biomarker signature published by Mellby et al. [74] and using their proprietary antibody platform, Immunovia is now developing the commercial assay IMMRay™ PanCan-d, which is currently being further validated in the prospective studies [111]. Our urinary biomarker panel comprising REG1B, LYVE1, TFF1 and the affiliated PancRISK score [112,113] are now being validated in a prospective study UroPanc [114] . A multi-analyte blood test that simultaneously evaluates the levels of eight cancer proteins and the presence of cancer gene mutations from circulating DNA, CancerSEEK^®^, screens for eight common cancers, including PDAC [115], and has already been commercialized. GRAIL is advocating its own Galleri test, which is based on cfDNA methylation signal and can detect over 50 different cancers [116]. Both platforms, however, suffer from lower SN in detecting stage I cancers, and there is a legitimate concern that the tests might be less specific in real-world conditions, where comorbidities may increase the risk of a false-positive result [117].

## 4. Conclusions

In summary, our systematic review demonstrates the current progress in the biomarker field and highlights several promising non-invasive PDAC biomarkers. Most of these, however, still require further validation prior to being deemed appropriate for translation into the clinical setting. All future studies should aim to improve methodological quality and the way of reporting. Novel biomarkers that have shown promising results should be further investigated on a larger number of specimens, as well as in a pre-diagnostic and prospective setting, to further demonstrate their real clinical utility in earlier detection of pancreatic adenocarcinoma.

## Figures and Tables

**Table 1:** Inclusion and Exclusion Criteria for study selection.

Inclusion Criteria
1. Pancreatic Ductal Adenocarcinoma
2. Non-invasive method of obtaining a liquid biopsy e.g., plasma, serum, urine, saliva, stool
3. Original data with reported AUC, SN and SP of a proposed biomarker
4. Human studies
5. Manuscripts from January 2010 until August 2020
**Exclusion Criteria**
1. No specification of what type of pancreatic cancer it was
2. Invasive procedures to obtain the biomarker e.g., tissue biopsy
3. No recorded data either of SN, SP and/or AUC for the tested biomarker
4. Biomarker used for a purpose other than detection e.g., prognostic biomarkers
5. Abstracts, Conference reports/writings, NHS reports, Review Articles

Abbreviations: AUC—Area Under the ROC (Receiver Operating Characteristic) Curve, SN—Sensitivity, SP—Specificity, NHS—National Health Service in UK.

**Table 2:** Key findings from included studies.

Reference	Specimen Type	Biomarker	Clinical Setting	Subjects	Sensitivity (%)	Specificity (%)	AUC
Gold et al., 2010 [37]	Serum	PAM4	68 PDAC, 19 HC	PDAC vs. HC	82.0	95.0	0.92 (0.84–0.97)
Joergensen et al., 2010 [38]	Serum	CA19-9 MMP-9 TIMP1	51 PDAC, 52 HC	PDAC vs. HC	86.0 58.8 47.1	73.0 34.6 69.2	0.84 (0.77–0.92) 0.50 (0.39–0.61) 0.64 (0.53–0.74)
Marten et al., 2010 [39]	Plasma	siC3b	157 PDAC, 38 HC	2–4mo prior radiologically defined recurrence	54.0	94.0	0.85
		siC3b		0–2mo prior radiologically defined recurrence	62.0	94.0	0.84
Brand et al., 2011 [17]	Serum	CA19-9 CA19-9 + ICAM-1 + OPG CA19-9 + CEA +TIMP-1	160 PDAC, 74 BPD, 107 HC	TS: PDAC vs. HC PDAC vs. BPD PDAC vs. HC	57,2 88.0 76.0	90.0 90.0 90.0	0.83 (0.81–0.86) 0.93 (0.91–0.95) 0.86
		CA19-9 CA19-9 + ICAM-1 + OPG CA19-9 + CEA +TIMP-1 CA19-9	173 PDAC, 70 BPD, 120 HC	VS: PDAC vs. BPD PDAC vs. HC PDAC vs. BPC PDAC vs. BPC	56.4 78.0 71.2 52.1	90.0 94.1 88.6 90.2	0.82 (0.78–0.86) 0.91 (0.88–0.95) 0.83 (0.88–0.89) 0.78 (0.74–0.83)
Park et al., 2012 [21]	Serum	Cathepsin D MMP-7 CA19-9 CA19-9 + Cathepsin D + MMP-7	109 PDAC, 40 HC, 30 CP	TS: PDAC vs. HC + CP	54.0 72.0 74.0 88.0	80.0 80.0 80.0 80.0	0.67 0.81 0.84 0.90 (p = 0.002)
		Cathepsin D MMP-7 CA19-9 CA19-9 + Cathepsin D + MMP-7	129 PDAC, 74 HC, 72 CP	VS: PDAC vs. HC + CP	53.0 65.0 78.0 89.0	79.0 79.0 84.0 77.0	0.65 0.77 0.88 0.91 (p = 0.002)
Capello et al., 2013 [40]	Serum	EZR-autoantibody	69 PDAC, 46 CP, 60 HC, 12 Aim, 50 Non-PDAC	PDAC vs. HC + CP + Aim	93.2	75.5	0.90
				PDAC vs. non-PDAC cancer	94.9	96.4	0.99
Gold et al., 2013 [41]	Serum	PAM4 CA19-9 PAM4 + CA19-9	298 PDAC, 120 BPD, 50 CP	PDAC vs. BPD	74.0 77.0 84.0	85.0 73.0 83.0	0.87 (p = 0.0001) 0.85 (p = 0.0257) 0.91
		PAM4 CA19-9 PAM4 + CA19-9		PDAC vs. CP	74.0 77.0 84.0	86.0 68.0 82.0	0.87 (p = 0.0001) 0.84 (p = 0.0073) 0.91
Kobayashi et al., 2013 [42]	Serum	Diagnostic Model * CA19-9 CEA Diagnostic Model * CA19-9 CEA	43 PDAC, 42 HC	TS: PDAC vs. HC	86.0 62.8 44.2	88.1 100.0 97.6	0.93 (0.86–0.97) 0.82 (0.70–0.90) 0.80 (0.69–0.88)
			9 PDAC (stage 0-IIB), 41 HC, 23 CP	VS: PDAC stage 0-IIB vs. HC + CP	77.8 55.6 44.4	78.1 85.9 79.7	0.76 (0.66–0.86) 0.79 (0.68–0.88) 0.67 (0.55–0.76)
Li et al., 2013 [43]	Serum	CA19-9 miR-1290 miR-146a miR-484	41 PDAC, 19 HC, 35 CP	PC vs. HC	71.0 88.0 78.0 76.0	90.0 84.0 79.0 63.0	0.86 0.96 (0.91–1.00) 0.82 (0.71–0.92) 0.78
		CA19-9 miR-1290 miR-146a miR-484		PDAC vs. CP	71.0 83.0 73.0 75.0	63.0 69.0 80.0 69.0	0.71 0.81 (0.71–0.91) 0.78 (0.68–0.89) 0.75
Zhao et al., 2013 [44]	Serum	miR -192	70 PDAC, 40 HC	PDAC vs. HC	76.0	55.0	0.63 (0.51–0.75)
Chung et al., 2014 [45]	Serum	sCD40L CA19-9 CEA	55 PDAC, 30 CP, 30 HC	VS: PDAC vs. CP vs. HC	80 80 68.9	85.5 72.7 60	0.88 0.78 0.70
Lee et al., 2014 [46]	Serum	CFB CA19-9 CFB + CA19-9	41 PDAC, 44 HC, 12 CP, 31 HCC, 22 CC, 35 GC	PDAC vs. non-PDAC	73.1 80.4 90.1	97.9 70.0 97.2	0.958 (0.956–0.959) 0.833 (0.829–0.837) 0.986 (p < 0.001)
Nolen et al., 2014 [47]	Serum	CA19-9 CA19-9 + CEA CA19-9 + CEA + Cyfra 21-1	343 PDAC, 227 HC	1–12 months Pre-diagnostic PDAC vs. HC	25.7 26.7 32.4	95.0 95.0 95.0	0.680 0.67 0.69
		CA19-9 CA19-9 + CEA CA19-9 + CEA + Cyfra 21-1		12–35 months Pre-diagnostic PDAC vs. HC	17.2 28.1 29.7	95.0 95.0 95.0	0.63 0.66 0.66
Ren et al., 2014 [48]	Serum	IL-11p	44 PDAC, 30 HC	PDAC vs. HC	97.7	70.0	0.901 (p < 0.001)
Schultz et al., 2014 [49]	Serum	Index 1 (miR-145, -150, -223, -636) Index 2 (miR-26b, -34a, -122, -126, -145, -150, -223, -505, -636, -885.5p) CA19-9 Index 1 + CA19-9 Index 2 + CA19-9	143 PDAC, 18 CP, 69 HC	DC: PDAC vs. HC + CP	85.0	73.0	0.88 (0.85–0.92)
					85.0 88.0 85.0 85.0	86.0 92.0 93.0 97.0	0.93 (0.89–0.96) 0.87 (0.82–0.92) 0.88 (0.83–0.93) 0.95 (0.92–0.98)
		Index 1 Index 2 CA19-9 Index 1 + CA19-9 Index 2 + CA19-9	180 PDAC, 199 HC	TS: PDAC vs. HC	85.0 85.0 86.0 85.0 85.0	64.0 85.0 99.0 95.0 98.0	0.86 (0.82–0.90) 0.93 (0.89–0.96) 0.90 (0.87–0.94) 0.93 (0.90–0.96) 0.97 (0.95–0.98)
		Index 1 Index 2 CA19-9 Index 1 + CA19-9 Index 2 + CA19-9	86 PDAC, 7 CP, 44 HC	VS: PDAC vs. HC + CP	85.0 85.0 79.0 85.0 85.0	45.0 51.0 88.0 88.0 86.0	0.83 (0.76–0.90) 0.81 (0.73–0.87) 0.89 (0.83–0.95) 0.93 (0.88–0.97) 0.92 (0.87–0.96)
Yang et al., 2014 [50]	Stool	miR-21 miR-155 miR -216 miR-216 + miR-21+ miR-155	30 PDAC, 15 HC	PDAC vs. HC	90.0 76.7 86.7 83.3	66.7 73.3 60.0 83.3	0.80 (0.68–0.92) 0.72 (0.58–0.86) 0.73 (0.60–0.86) 0.87 (0.77–0.96)
Zhang et al., 2014 [51]	Serum	CA19-9 + Albumin + CRP + IL-8 CA19-9 CA19-9 + Albumin + CRP + IL-8 CA19-9	163 PDAC (77 Early stage I-II), 109 BC, 200 HC	All stage PDAC vs. HC All stage PDAC vs. HC Early stage PDAC vs. HC Early stage PDAC vs. HC	99.4 80.6 96.1 72.7	90.0 90.0 90.0 90.0	0.98 (0.97–1.00) 0.85 (0.80–0.90) 0.97 (0.95–1.00) 0.83 (0.75–0.90)
		CA19-9 + CO2 +CRP + IL-6 CA19-9 CA19-9 + CO2 +CRP + IL-6 CA19-9		All stage PDAC vs. BC All stage PDAC vs. BC Early stage PDAC vs. BC Early stage PDAC vs. BC	74.2 53.4 75.3 40.3	90.0 90.0 90.0 90.0	0.89 (0.86–0.93) 0.75 (0.69–0.81) 0.87 (0.82–0.93) 0.69 (0.61–0.78)
Debernardi et al., 2015 [52]	Urine	miR-143 miR-223 miR-30e miR-143 + miR-30e	6 PDAC (stage I), 26 HC	Stage I PDAC vs. HC	83.3 83.3 83.3 83.3	88.5 76.9 80.8 96.2	0.86 (0.70–1.00) 0.80 (0.59–1.00) 0.85 (0.67–1.00) 0.92 (0.79–1.00)
Han et al., 2015 [53]	Serum	Dickkopf-1 (DKK1) CA19-9	140 PDAC, (62 Early stage I-II), 48 HC, 18 BPT, 26 CP	PDAC vs. HC + BPT + CP	89.3 73.6	79.4 83.7	0.92 (0.88–0.95) 0.85 (0.80–0.90)
		Dickkopf-1 (DKK1) CA19-9		PDAC vs. BPT + CP	89.3 73.6	72.7 81.8	0.89 (0.83–0.95) 0.83 (0.77–0.89)
		Dickkopf-1 (DKK1) CA19-9		Early-PDAC vs. HC + BPT + CP	85.5 64.5	79.3 83.7	0.89 (0.84–0.94) 0.81 (0.74–0.89)
		Dickkopf-1 (DKK1) CA19-9		Early-PDAC vs. BPT + CP	85.5 64.5	72.7 81.8	0.85 (0.78–0.93) 0.78 (0.70–0.87)
Melo et al., 2015 [54]	Serum	GPC1 + crExos CA19-9	190 PDAC, 100 HC, 26 BPD	DS: PDAC vs. HC + BPD	100.0 76.8	100.0 64.3	1.00 (0.99–1.00) 0.74 (0.70–0.83)
		GPC1 + crExos GPC1 (ELISA)	56 PDAC, 20 HC, 6 BPD	VS: PDAC vs. HC + BPD	100.0 82.1	100.0 75.0	1.00 (0.96–1.00) 0.78 (0.68–0.87)
Radon et al., 2015 [55]	Urine	LYVE1 REG1A TFF1 LYVE1 + REG1A + TFF1 + (Creatinine +Age)	143 PDAC (stage I-IV), 59 HC	TS: PDAC vs. HC	76.9 62.2 72.7 76.9	88.1 94.9 59.3 89.8	0.85 (0.80–0.90) 0.82 (0.77–0.88) 0.69 (0.61–0.77) 0.89 (0.85–0.94)
		LYVE1 REG1A TFF1 LYVE1 + REG1A + TFF1 + (Creatinine +Age)	56 PDAC (stage I-II), 61 HC		67.9 75.0 78.6 82.1	91.8 68.9 52.5 88.5	0.84 (0.77–0.91) 0.75 (0.66–0.84) 0.70 (0.60–0.79) 0.90 (0.84–0.96)
		LYVE1 + REG1A + TFF1 + (Creatinine +Age)	49 PDAC (stage I-IV), 28 HC	VS: PDAC vs. HC	75.5	100	0.92 (0.84–1.00)
		LYVE1 + REG1A + TFF1 + (Creatinine +Age)	56 PDAC (stage I-II), 61 HC		80.0	76.9	0.93 (0.84–1.00)
		Plasma CA19-9 Panel (LYVE1 + REG1A + TFF1) Panel + Plasma CA19-9	71 PDAC (stage I-II), 28 HC	Exploratory Comparison	83.1 93.0 94.4	92.9 92.9 100	0.88 (0.81–0.95) 0.97 (0.95–1.00) 0.99 (0.98–1.00)
Ankeny et al., 2016 [56]	Serum	CTCs and KRAS mutation analysis	72 PDAC, 28 non-adenocarcinoma	PDAC vs. non-adenocarcinoma	78.0	96.4	0.87 (0.80–0.94)
Guo et al., 2016 [57]	Serum	DTNBP1 CA19-9	250 PDAC, 70 CP, 80 BBO, 150 HC	PDAC vs. BBO + CP + HC	81.9 76.3	84.7 52.5	0.85 (0.81–0.89) 0.74 (0.70–0.78)
		DTNBP1 CA19-9		PDAC vs. CP	73.9 66.3	78.9 73.2	0.80 (0.75–0.86) 0.69 (0.63–0.75)
		DTNBP1 CA19-9		PDAC vs. BBO	82.3 53.8	84.0 49.4	0.85 (0.80–0.89) 0.59 (0.53–0.65)
Henriksen et al., 2016 [58]	Plasma	(Model13): age >65+ BMP3+ RASSF1A+ BNC1+ MESTv2+ TFPI2+ APC+ SFRP1 + SFRP2	95 PDAC, 97 CP, 27 “screened negative”	PDAC vs. screened negative + CP	73.0	83.0	0.86 (0.81–0.91)
Sogawa et al., 2016 [59]	Serum	C4BPA CA19-9 CEA C4BPA + CA19-9	66 PDAC, 40 HC, 20 CP	PDAC vs. HC + CP	67.3 71.2 34.6 86.4	95.4 95.4 95.4 95.4	0.86 (p < 0.001) 0.85 0.77 0.93
		C4BPA CA19-9 CEA	18 PDAC (stage I-II), 40 HC, 20 CP	PDAC stage I-II vs. HC + CP	50.0 22.2 22.2	95.4 95.4 95.4	0.91 (p < 0.001) 0.74 0.87
Yoneyama et al., 2016 [60]	Serum	CA19-9 IGFBP2 IGFBP3	38 PDAC (stage I-II), 65 HC	Stage I-II PDAC vs. HC	60.5 68.4 76.3	92.3 67.7 70.7	0.84 (0.75–0.93) 0.71 (0.60–0.81) 0.77 (0.67–0.86)
Balasenthil et al., 2017 [61]	Plasma	CA19-9	55 PDAC (stage IA/ IB-IIA), 61HC	Stage IA/ IB-IIA vs. HC	71.0	61.0	0.74 (0.64–0.84)
		TNC + TFP1 + CA19-9	55 PDAC (stage IA/ IB-IIA), 62 CP	Stage IA/ IB-IIA vs. CP	73.0	82.0	0.79 (0.70–0.87)
		CA19-9 TNC + TFP1 + CA19-9			71.0 73.0	44.0 71.0	0.69 (0.58–0.79) 0.75 (0.65–0.84)
Yang et al., 2017 [62]	Plasma	EGFR EPCAM HER2 MUC1 GPC1 WNT2 GRP94 B7-H3 EGFR + EPCAM + HER2 + MUC1 EGFR + EPCAM + GPC1 + WNT2 EGFR + EPCAM + MUC1 + GPC1 + WNT2 EGFR + EPCAM + HER2 + MUC1 + GPC1 + WNT2	22 PDAC, 10 HC	TS: PDAC vs. HC	73 73 59 36 55 77 73 50 91 100 100 100	100 100 90 100 60 90 70 100 100 100 100 100	0.90 (0.79–1) 0.88 (0.77–0.99) 0.72 (0.55–0.89) 0.66 (0.48–0.84) 0.48 (0.28–0.67) 0.84 (0.71–0.96) 0.73 (0.55–0.90) 0.75 (0.58–0.93) 0.99 (0.97–1) 1.0 1.0 1.0
		EGFR EPCAM HER2 MUC1 GPC1 WNT2 GRP94 EGFR + EPCAM + HER2 + MUC1 EGFR + EPCAM + GPC1 + WNT2 EGFR + EPCAM + MUC1 + GPC1 + WNT2 EGFR + EPCAM + HER2 + MUC1 + GPC1 + WNT2	22 PDAC, 8 CP, 5 BPD, 8 other abdominal indications	VS: PDAC vs. CP vs. BPD vs. other abdominal indications	59 45 59 36 82 64 55 86 82 86 95	76 95 85 90 52 76 71 86 90 81 81	67 (51–81) 70 (54–83) 72 (56–85) 63 (47–77) 67 (51–81) 70 (54–83) 63 (47–77) 86 (72–95) 86 (72–95) 84 (69–93) 88 (75–96)
Capello et al., 2017 [63]	Serum/ Plasma	CA19-9 TIMP1 + LRG1 + CA19-9 TIMP1 + LRG1 + CA19-9 (“OR” Rule) CA19-9	39 early stage PDAC, 82 HC	TS: early stage PDAC vs. HC	53.8 66.7 72.6 84.9	95.0 95.0 95.0 95.0	0.82 (0.74–0.91) 0.89 (0.82–0.96) 0.88 (0.81–0.96) 0.95 (0.92–0.98)
		CA19-9 TIMP1 + LRG1 + CA19-9	73 early stage PDAC, 60 HC	VS: early stage PDAC vs. HC	84.9 28.8	95.0 95.0	0.96 (0.89–1.00) 0.82 (0.74–0.91)
Hussein et al., 2017 [64]	Serum	miR-22-3p miR-642b-3p miR-885-5p CA19-9	35 PDAC (33 early stage, 2 late stage) 15 HC	PDAC vs. HC	97.1 100 100 91.4	93.3 100 100 100	0.94 (p < 0.001) 1.00 (p < 0.001) 1.00 (p < 0.001) 0.92 (p < 0.001)
Kaur et al., 2017 [65]	Serum	MUC5AC CA19-9	70 PDAC (stage I or II), 43 CP, 35 HC, 30 BC	Early PDAC vs. HC	83.0 56.0	80.0 95.0	0.87 (0.79–0.95) 0.72 (0.59–0.84)
		MUC5AC CA19-9		Early PDAC vs. BC	67.0 48.0	87.0 89.0	0.85 (0.76–0.93) 0.71 (0.59–0.83)
		MUC5AC CA19-9		Early PDAC vs. CP	83.0 48.0	77.0 86.0	0.84 (0.76–0.92) 0.62 (0.50–0.74)
		MUC5AC CA19-9 MUC5AC + CA19-9		PDAC vs. HC + BC + CP	89.0 79.0 83.0	70.0 43.0 83.0	0.88 (0.83–0.93) 0.61 (0.54–0.68) 0.91 (0.86–0.95)
Kim et al., 2017 [66]	Plasma	CA19-9 (≥55) THBS2 (36ng/mL cut-off) CA19-9 + THBS2	58 (stage I-II, phase 2a), 80 HC	PDAC stage I or II vs. HC	69.0 33.0 74.1	100 96.0 96.3	0.85 (0.80–0.89) 0.83 (0.78–0.89) 0.95 (0.92–0.98)
		CA19-9 THBS2 CA19-9 + THBS2	88 (stage I-II, phase 2b), 140 HC		77.7 58.4 88.3	98.6 93.6 92.9	0.83 (0.79–0.97) 0.89 (0.85–0.92) 0.96 (0.94–0.98)
Lai et al., 2017 [67]	Plasma	CA19-9 miR-10b miR-21 miR-30c miR-106b miR-20a miR-181a miR-483 miR-let7a miR-122	29 PDAC, 6 HC	PDAC vs. HC	86.0 100.0 86.0 100.0 97.0 93.0 97.0 66.0 93.0 100.0	100.0 100.0 100.0 100.0 100.0 100.0 100.0 67.0 100.0 67.0	0.92 (p < 0.001) 1.00 (p < 0.001) 0.95 (p < 0.001) 1.00 (p < 0.001) 0.98 (p < 0.001) 0.99 (p < 0.001) 0.97 (p < 0.001) 0.67 (p = 0.20) 0.99 (p < 0.001) 0.89 (p = 0.003)
Park et al., 2017 [68]	Serum	LRG1 + TTR + CA19-9 CA19-9 LRG1 + TTR + CA19-9 CA19-9 LRG1 + TTR + CA19-9 CA19-9 LRG1 + TTR + CA19-9 CA19-9 LRG1 + TTR + CA19-9 CA19-9	80 PDAC (50 stage I-II) (29 CA19-9 negative PDAC), 68 HC, 21 BPD, 52 Thyroid Ca, 52 Breast Ca, 45 Colorectal Ca)	PDAC vs. HC + BPC (*n* = 89) PDAC stage I-II vs. HC + BPC (*n* = 89) PDAC vs. Other Cancers (*n* = 149) PDAC vs. BPD CA19-9 negative PDAC (*n* = 29) vs. HC + BPC	82.5 72.5 76.0 64.0 82.5 72.5 82.5 72.5 51.7 24.1	92.1 88.8 92.1 88.8 83.9 87.9 85.7 81.0 92.1 88.8	0.93 (p < 0.01) 0.83 0.91 (p < 0.01) 0.79 0.90 (p < 0.001) 0.80 0.90 0.81 0.83 (p < 0.001) 0.52
Schott et al., 2017 [69]	Serum	HYAL2 Methylation	82 PDAC, 191 HC 60 PDAC (stage I-II), 191 HC	PDAC vs. HC PDAC stage I-II vs. HC	75.6 66.7	93.7 95.3	0.92 (0.88–0.96) 0.93 (0.89–0.98)
Arasaradnam et al., 2018 [70]	Urine	Volatile organic compounds	4 PDAC stage I, 5 stage IIA, 35 stage IIB, 24 stage III, 12 stage IV, 81 HC	TS: PDAC vs. HC VS: PDAC vs. HC PDAC stage I -II vs. HC PDAC stage I -II vs. PDAC stage III-IV	0.91 0.90 0.91 0.82	0.83 0.81 0.78 0.89	0.92 (0.88–0.96) 0.92 (0.85–0.98) 0.89 (0.83–0.94) 0.92 (0.86–0.97)
Dong et al., 2018 [71]	Serum	CA19-9 POSTN CA242 CA19-9 + POSTN CA19-9 + CA242 POSTN+ CA242 CA19-9 + POSTN+ CA242	30 PDAC (early stage), 68 PDAC (late stage), 32 BPC, 37 HC, 27 PDAC (CA19-9 negative)	TS: HC vs. early PDAC	96.7 70.0 81.1 93.3 90.0 83.3 96.7	83.8 75.7 81.1 94.6 94.6 86.5 94.6	0.94 (0.86–0.99) 0.78 (0.66–0.87) 0.89 (0.79–0.95) 0.97 (0.90–1.00) 0.96 (0.88–0.99) 0.90 (0.80–0.96) 0.98 (0.90–1.00)
		CA19-9 POSTN CA242 CA19-9 + POSTN CA19-9 + CA242 POSTN+ CA242 CA19-9 + POSTN+ CA242		BPC vs. all PDAC	85.7 64.3 58.1 84.7 75.5 67.4 84.7	81.3 87.5 87.5 90.6 90.6 96.9 90.6	0.88 (0.82–0.93) 0.81 (0.74–0.88) 0.78 (0.70–0.85) 0.93 (0.88–0.97) 0.89 (0.83–0.94) 0.87 (0.80–0.92) 0.94 (0.88–0.97)
		CA19-9 POSTN CA242 CA19-9 + POSTN CA19-9 + CA242 POSTN+ CA242 CA19-9 + POSTN+ CA242	38 PDAC (early stage), 77 PDAC (late stage), 43 BPC; 37 HC, 29 PDAC (CA19-9 negative)	BPC vs. early PDAC	86.7 53.3 83.3 96.7 90.0 56.7 96.7	81.3 87.5 62.5 75.0 78.1 96.9 75.0	0.88 (0.77–0.95) 0.74 (0.61–0.84) 0.78 (0.66–0.88) 0.90 (0.80–0.96) 0.90 (0.79–0.96) 0.80 (0.68–0.89) 0.92 (0.82–0.97)
		CA19-9 POSTN CA242 CA19-9 + POSTN CA19-9 + CA242 POSTN+ CA242 CA19-9 + POSTN+ CA242		VS: HC vs. early PDAC	86.8 63.2 57.9 86.8 86.8 79.0 92.1	94.6 78.4 100 97.3 97.3 94.6 97.3	0.94 (0.86–0.98) 0.78 (0.66–0.86) 0.83 (0.73–0.91) 0.95 (0.88–0.99) 0.97 (0.90–1.00) 0.92 (0.84–0.97) 0.98 (0.92–1.00)
		CA19-9 POSTN CA242 CA19-9 + POSTN CA19-9 + CA242 POSTN+ CA242 CA19-9 + POSTN+ CA242		BPC vs. all PDAC	84.4 77.4 60.0 83.5 77.4 84.4 80.0	81.4 79.1 93.0 93.0 88.4 83.7 97.7	0.88 (0.82–0.93) 0.82 (0.76–0.88) 0.79 (0.72–0.85) 0.92 (0.87–0.96) 0.90 (0.84–0.94) 0.89 (0.83–0.93) 0.93 (0.88–0.96)
		CA19-9 POSTN CA242 CA19-9 + POSTN CA19-9 + CA242 POSTN+ CA242 CA19-9 + POSTN+ CA242		BPC vs. early PDAC	86.8 65.8 57.9 65.8 76.3 76.3 83.6	79.1 79.1 93.0 93.0 93.0 86.1 88.4	0.87 (0.78–0.93) 0.72 (0.61–0.82) 0.77 (0.66–0.85) 0.84 (0.75–0.92) 0.92 (0.83–0.97) 0.84 (0.74–0.91) 0.90 (0.81–0.96)
Guo et al., 2018 [72]	Serum	SNHG15	171 PDAC, 59 HC	PDAC vs. HC	68.3	89.6	0.73 (p < 0.01)
Lewis et al., 2018 [73]	Whole blood, plasma, serum	GPC1 + CD63	20 PDAC 6 BPD	PDAC vs. BPD	81	70	0.79 (0.99–1.00)
Mellby et al., 2018 [74]	Plasma	Panel of 29 biomarkers	15 PDAC stage I, 75 stage II, 15 stage III, 38 stage IV 57 CP, 20 IPMN, 219 HC	TS 2: PDAC stage I-II vs. HC	95	94	0.96 (0.94–0.98)
				VS (USA cohort): PDAC stage I-II vs. HC	93	95	0.96 (0.94–0.98)
Traeger et al., 2018 [75]	Serum	miRNA-205 CA19-9 miRNA-205 +CA19-9	47 PDAC, 16 CP, 5 IPMN, 17 BPC, 17 HC	PDAC vs. non-PDAC	0.643 0.810 0.867	0.684 0.768 0.933	0.70 (0.548–0.789) 0.79 (0.698–0.887) 0.89 (0.782–0.995)
Zhou et al., 2018 [76]	Serum	GPC1 CA19-9	156 PDAC, 20 BPT, 16 CP, 163 HC	PDAC vs. HC + BPT + CP	76.92 82.69	70.85 93.97	0.80 (0.749–0.841) 0.91 (0.868–0.947)
		GPC1 CA19-9		PDAC vs. HC	76.92 82.69	70.55 97.55	0.81 (0.763–0.856) 0.91 (0.875–0.953)
		GPC1 CA19-9		Early PDAC vs. HC + BPT + CP	68.06 79.17	70.85 93.97	0.76 (0.695–0.816) 0.88 (0.816–0.946)
		GPC1 CA19-9		Early PDAC vs. HC	68.06 79.17	70.55 97.55	0.77 (0.705–0.830) 0.89 (0.824–0.953)
Berger et al., 2019 [77]	Plasma	CA19-9 THBS2 cfDNA CA19-9 + THBS2 THBS2 + CA19-9 + cfDNA	52 PDAC, 15 IPMN, 32 CP	TS: PDAC vs. IPMN vs. CP	55 41 32–86 ^1^ 73 41–86 ^1^	91 96 70–100 ^1^ 91 78–96 ^1^	0.80 0.73 0.90 0.87 0.94
		CA19-9 THBS2 cfDNA CA19-9 + THBS2 THBS2 + CA19-9 + cfDNA		VS: PDAC vs. IPMN vs. CP	63 50 43–80 ^1^ 80 50–93 ^1^	96 96 79–96 ^1^ 96 92–96 ^1^	0.70 0.63 0.81 0.78 0.88
Eissa et al., 2019 [78]	Plasma	ADAMTS1	39 PDAC, 95 HC	PDAC vs. HC	87.2	95.8	0.91 (0.77–0.90)
		BNC1			64.1	93.7	0.79 (0.63–0.78)
		ADAMTS1 and/or BNC1			97.4	91.6	0.95 (0.71–0.86)
Fahrmann et al., 2019 [79]	Plasma	AcSperm+ DAS + indole-derivative+ LysoPC(18:0) + LysoPC(20:3)	29 PDAC, 10 HC	TS: PDAC vs. HC	69.0	99.0	0.90 (0.818–0.989)
		AcSperm+ DAS + indole-derivative+ LysoPC(18:0) + LysoPC(20:3)	39 Resectable PDAC, 82 HC	VS: PDAC vs. HC	66.7	43.3	0.89 (0.828–0.996)
		Indole-derivative LysoPC(18:0) LysoPC(20:3) AcSperm DAS			23.1 51.3 48.7 33.3 51.3	11.3 26.3 11.3 27.5 27.5	0.73 (0.631–0.822) 0.84 (0.764–0.920) 0.84 0.757–0.925) 0.76 (0.659–0.852) 0.80 (0.712–0.890)
Yu et al., 2019 [80]	Plasma	d-signature: EV long RNA (FGA, KRT19, HIST1H2BK, ITIH2, MARCH2, CLDN1, MAL2 and TIMP1)	284 PDAC, 100 CP, 117 HC	PDAC vs. CP vs. HC d-signature with CA19-9	93.68	91.57	0.936 (0.889–0.983)
Takahashi et al., 2019 [81]	Serum	Circulating EV-encapsulated HULC	20 PDAC, 22 IPMN, 21 HC	PDAC vs. IPMN vs. HC	80	92.1	0.92
Wei et al., 2019 [82]	Plasma	Vimetin-positive CTCs	100 PDAC, 16 IPMN, 30 HC	PDAC vs. IPMN vs. HC	65	100	0.968
Yang et al., 2020 [83]	Plasma	EV miRNAs and mRNAs, cfDNA, ccfDNA KRAS G12D/V/R mutations and CA19-9	30 CP + BPC, 49 PDAC, 57 HC	PDAC vs. non-PDAC vs. HC	88	95	0.95 (p= 0.103)

Abbreviations: Aim: autoimmune diseases; BBO: benign biliary obstruction; BBP: benign biliary pathology; BCT: benign cystic tumor; BPD: benign pancreatic disease; BPT: benign pancreatic tumor; CC: cholangiocarcinoma; ccfDNA: circulating cell-free DNA; cf: circulating free; CP: chronic pancreatitis; CTC: circulating tumor cell; DC: discovery cohort; EV: extracellular vesicles; GC: gastric cancer; HC: healthy control; HCC: hepatocellular carcinoma; HULC: highly upregulated in liver cancer; IPMN: intraductal papillary mucinous neoplasm; PC: prospective cohort; PDAC: pancreatic ductal adenocarcinoma; TS: training set; VS: validation set. Note: ^1^ Depending on the concentration of cfDNA.

## Appendix A

**Table A1:** Graphical summary of the QUADAS-2 scores for each of the 49 studies.

Nr.	Study	Risk of Bias			Applicability Concerns			Score
		Patient Selection	Index Test	Reference Standard	Patient Selection	Index Test	Reference Standard	
**1**	Gold et al., 2010 [37]	☺	?	?	☺	☺	☺	Low
**2**	Joergensen et al., 2010 [38]	☺	?	☺	☺	☺	☺	Low
**3**	Marten et al., 2010 [39]	☹	?	☺	☹	?	☺	High
**4**	Brand et al., 2011 [17]	☺	☺	?	☹	☺	☺	Low
**5**	Park et al., 2012 [21]	☺	?	☺	☺	☺	☺	Low
**6**	Capello et al., 2013 [40]	☺	☺	☺	☹	☺	☺	Low
**7**	Gold et al., 2013 [41]	☺	?	☺	☺	☺	☺	Low
**8**	Kobayashi et al., 2013 [42]	☺	☹	☺	☹	☺	☺	Low
**9**	Li et al., 2013 [43]	☺	☺	☺	☺	☺	☺	Low
**10**	Zhao et al., 2013 [44]	☹	☹	?	☺	☺	☺	High
**11**	Chung et al., 2014 [45]	☺	☺	☺	☺	☺	☺	Low
**12**	Lee et al., 2014 [46]	☺	☺	☺	☹	☺	☺	Low
**13**	Nolen et al., 2014 [47]	☺	☺	☺	☹	☺	☺	Low
**14**	Ren et al., 2014 [48]	☺	☺	☺	☺	☺	☺	Low
**15**	Schultz et al., 2014 [49]	☺	?	☺	☺	☺	☺	Low
**16**	Yang et al., 2014 [50]	☺	☹	?	☹	?	☺	High
**17**	Zhang et al., 2014 [51]	☺	?	☺	☺	☺	☺	Low
**18**	Debernardi et al., 2015 [52]	☹	?	?	☺	☺	☺	High
**19**	Han et al., 2015 [53]	☺	☹	☺	☺	☺	☺	Low
**20**	Melo et al., 2015 [54]	☺	?	☺	☺	☺	☺	Low
**21**	Radon et al., 2015 [55]	☺	?	☺	☺	☺	☺	Low
**22**	Ankeny et al., 2016 [56]	☺	☹	?	☺	?	☺	High
**23**	Guo et al., 2016 [57]	☺	☺	☺	☹	☺	☺	Low
**24**	Henriksen et al., 2016 [58]	☺	☹	?	☺	☺	☺	Low
**25**	Sogawa et al., 2016 [59]	☺	☹	☺	☺	☺	☺	Low
**26**	Yoneyama et al., 2016 [60]	☺	?	☺	☹	☺	☺	Low
**27**	Balasenthil et al., 2017 [61]	☺	☺	☺	☺	☺	☺	Low
**28**	Yang et al., 2017 [62]	☺	?	☺	☺	?	☺	Low
**29**	Capello et al., 2017 [63]	☺	?	☺	☺	☺	☺	Low
**30**	Hussein et al., 2017 [64]	☹	☹	☺	☹	☺	☺	High
**31**	Kaur et al., 2017 [65]	☺	☺	☺	☺	☺	☺	Low
**32**	Kim et al., 2017 [66]	☺	☺	☺	☺	☺	☺	Low
**33**	Lai et al., 2017 [67]	☹	☹	☺	☹	☺	☺	High
**34**	Park et al., 2017 [68]	☺	☹	☺	☺	☺	☺	Low
**35**	Schott et al., 2017 [69]	☹	☹	?	☺	☺	☺	High
**36**	Arasaradnam et al., 2018 [70]	☹	?	?	☺	☺	☺	High
**37**	Dong et al., 2018 [71]	☺	?	☺	☺	☺	☺	Low
**38**	Guo et al., 2018 [72]	☹	☹	?	☺	☺	☺	High
**39**	Mellby et al., 2018 [74]	☺	?	☺	☺	☺	☺	Low
**40**	Traeger et al., 2018 [75]	☺	☹	☺	☺	☺	☺	Low
**41**	Zhou et al., 2018 [76]	☺	☹	☺	☹	?	☺	High
**42**	Berger et al., 2019 [77]	☺	☺	☺	☺	☺	☺	Low
**43**	Eissa et al., 2019 [78]	☺	☹	?	☺	☺	☺	Low
**44**	Fahrmann et al., 2019 [79]	☺	☺	☺	☺	☺	☺	Low
**45**	Lewis et al., 2019 [73]	☺	?	?	☹	?	☺	High
**46**	Yu et al., 2019 [80]	☺	☹	☺	☺	☺	☺	Low
**47**	Takahashi et al., 2019 [81]	☺	☹	?	☺	?	☺	High
**48**	Wei et al., 2019 [82]	☺	☹	☺	☺	?	☺	Low
**49**	Yang et al., 2020 [83]	☺	☺	☺	☺	?	☺	Low

☺ Low Risk; ☹ High Risk; ? Unclear Risk.
